# Tumour budding-based grading as independent prognostic biomarker in HPV-positive and HPV-negative head and neck cancer

**DOI:** 10.1038/s41416-023-02240-y

**Published:** 2023-04-12

**Authors:** Fabian Stögbauer, Susanne Beck, Iordanis Ourailidis, Jochen Hess, Christopher Poremba, Maren Lauterbach, Barbara Wollenberg, Anna Maria Stefanie Buchberger, Moritz Jesinghaus, Peter Schirmacher, Albrecht Stenzinger, Wilko Weichert, Melanie Boxberg, Jan Budczies

**Affiliations:** 1 Institute of Pathology, School of Medicine, Technical University of Munich (TUM), 81675 Munich, Germany; 2 University of Heidelberg, Institute of Pathology, Im Neuenheimer Feld 224, 69120 Heidelberg, Germany; 3 Section Experimental and Translational Head and Neck Oncology, Department of Otolaryngology, Head and Neck Surgery, University Heidelberg, Im Neuenheimer Feld 400, 69120 Heidelberg, Germany; 4 Research Group Molecular Mechanisms of Head and Neck Tumors, German Cancer Research Center (DKFZ), Im Neuenheimer Feld 280, 69120 Heidelberg, Germany; 5 Pathologie München-Nord, 80992 Munich, Germany; 6 Department of Otorhinolaryngology Head and Neck Surgery, University Hospital Klinikum Rechts der Isar, Ismaningerstr. 22, 81675 Munich, Germany; 7 Institute of Pathology, University Hospital Marburg, Baldingerstraße, 35043 Marburg, Germany; 8 German Cancer Consortium (DKTK), Munich and Heidelberg partner sites, Munich and Heidelberg, Germany

**Keywords:** Prognostic markers, Surgical oncology, Prognostic markers

## Abstract

**Background:**

The prognostic significance of tumour budding (TB) and minimal cell nest size (MCNS) was shown in human papillomavirus (HPV)-negative head and neck squamous cell carcinomas (HNSCC). However, the optimisation of cutpoints, the prognostic impact in HPV-positive HNSCC, and the comparison with other histopathological grading systems are insufficiently investigated.

**Methods:**

TB and MCNS were analysed digitally in 1 and 10 high-power fields (HPF) of 331 HPV-positive and HPV-negative cases from TCGA. Optimising the cutpoints a new cellular dissociation grading (CDG) system was defined and compared to the WHO grading and the Brandwein–Gensler (BG) risk model.

**Results:**

The two-tiered CDG system based solely on TB yielded optimal prognostic stratification with shortened overall survival for CDG-high cases. Optimal cut-offs were two buds (1 HPF) and six buds (10 HPF), respectively. Analysing MCNS did not add prognostic significance to quantifying TB. CDG was a significant prognostic marker in HPV-negative and HPV-positive tumours and prognostically superior to the WHO and BG systems. High CDG was associated with clinically occult lymph-node metastases.

**Conclusions:**

The most comprehensive study of TB in HNSCC so far confirmed its prognostic impact in HPV-negative tumours and for the first time in HPV-positive tumours. Further studies are warranted to evaluate its applicability for therapy guidance in HNSCC.

## Background

Squamous cell carcinomas of the head and neck (HNSCC) represent the seventh most common cancer entity worldwide with an annual incidence of approximately 900,000 cases [1, 2]. While HNSCC of the oral cavity, larynx and hypopharynx are frequently associated with tobacco smoking and/or alcohol abuse, about 30–80% of oropharyngeal tumours are positive for high-risk human papillomaviruses (HPV) [3–5]. Despite recent advances in cancer medicine, the long-term survival of patients with HNSCC remains poor [6–8]. Established biomarkers for treatment stratification (e.g. the World Health Organization (WHO) histopathologic grading system) lack prognostic power and other reliable prognostic markers, besides HPV status, are currently not established in clinical practice [9–11].

Tumour budding (TB) has emerged as a promising tissue-based biomarker in various solid tumour entities [12–15]. TB is defined as the detachment of tumour cell clusters consisting of up to four tumour cells from the main tumour mass [16]. It is supposed to be the morphologic manifestation of (partial) epithelial-mesenchymal transition establishing the invasive potential of tumours, inducing metastatic spread and subsequently causing poor prognosis [17–19]. In previous studies, the association of TB with poor overall survival in patients with oral, laryngeal and hypopharyngeal HPV-negative squamous cell carcinomas was demonstrated and its superiority over WHO tumour grading was shown [9, 12, 13, 15, 19–23]. However, despite the promising prognostic significance of TB there are several obstacles that need to be overcome before applying TB in clinical decision making including staining techniques (H&E or immunohistochemistry), qualitative or quantitative assessment methods, cutpoint values and area of examination [24].

To pave the way for an optimised and standardised evaluation of TB in HNSCC, we systematically analysed the TCGA-HNSC cohort. To the best of our knowledge, the current study is the first evaluation of TB in this cohort and the prognostic study of TB in HNSCC with the largest sample size. We aimed to identify optimal TB cutpoints for prognostic patient stratification, to develop an optimised cellular dissociation grading (CDG) system, and to compare the CDG system with two established histopathological grading systems, the WHO grading and the Brandwein–Gensler (BG) risk model [11, 25]. WHO grading was primarily defined for HPV-negative tumours and TB as well as BG risk have so far only been systematically evaluated in HPV-negative tumours. By contrast, as an essential number of patients with HPV-positive tumours suffer from recurrent disease and tumour-related death, prognostic biomarkers are of particular interest in this subgroup of patients [26]. Thus, we evaluated and compared all three histopathological grading systems in both the HPV-negative and the HPV-positive patients.

## Methods

### TCGA cohort

The TCGA-HNSC cohort included a total of 528 patients who were treated for HNSCC [27, 28]. Digitised H&E-stained diagnostic slides of 471 cases were available from the GDC Data Portal (https://portal.gdc.cancer.gov). A total of 331 tumours consisting of conventional, basaloid, verrucous, and papillary HNSCC were included into the study after exclusion of 140 cases. Cases were excluded due to the following reasons: small biopsy specimen precluding the analysis of 10 HPF, a different tumour entity, sarcomatoid histomorphology, not enough tumour on slide, inferior scan quality, exposed carcinoma without relation to surrounding stroma precluding the analysis of TB, duplicates and a history of neoadjuvant treatment. HPV status was determined by investigating the tumour DNA with a PCR based assay interrogating 16 HPV types (16, 18, 31, 33, 35, 39, 45, 51, 52, 56, 58, 59, 66, 68, 73, and 90) as previously described [27]. Tumours were classified as HPV+ when the test result was positive, as HPV- when the test result was negative and excluded from the analysis when the test result was indeterminate. The clinicopathological characteristics of the study cohort are shown in Supplemental Table S1.

### Histomorphologic analysis

Diagnostic slides were evaluated by three experienced pathologists (MB, FS, CP) using Aperio ImageScope ×64 (version 12.4.0.7018; Leica Biosystems GmbH, Nussloch, Germany) and a standard monitor (Fujitsu B24T-7, Fujitsu Limited, Tokyo, Japan, resolution 1920 ×1080). The raters were blinded with respect to the clinicopathological data. Analyses were conducted independently by raters and ambiguous cases were discussed until a consensus was reached.

According to the current WHO classification, subtyping of HNSCC into conventional, basaloid, papillary and verrucous tumours was conducted and histopathologic grading was applied for HPV-negative tumours (well, moderately, poorly differentiated). This grading is based on determination of the histopathologic differentiation in terms of “similarity” to healthy squamous epithelium as described by Broder in the 1920s [11, 29]. Although grading of (at least oropharyngeal) HPV-positive HNSCC is not established [11], we determined a histopathologic grade in analogy to HPV-negative tumours to enable the calculation of correlations between WHO grading and TB.

Perineural invasion was defined as described before [25], lymphangioinvasion was stated as present when carcinoma cells were detected within lymphatic spaces.

**Fig. 1:**
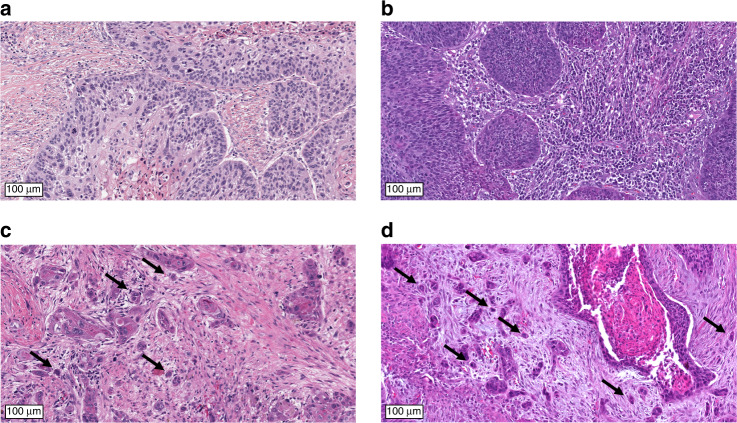
Evaluation of tumour budding (TB) in H&E-stained tissue sections of HNSCC. While tumour budding (TB) was absent in the TCGA cases **a** and **b**, strong TB was observed in the TCGA cases **c** and **d**. Exemplary budding foci are highlighted by arrows.

Tumour buds were defined as clusters of up to four tumour cells separating from the tumour mass and infiltrating into surrounding stroma (Fig. 1) [20, 21, 30, 31]. The whole tumour area was evaluated and the focus with the highest amount of tumour buds was determined. One digital high-power field (HPF, 97,464 µm^2^, corresponding to a field diameter of 0.35 mm in light microscopy) within this focus was analysed at high-power magnification. TB was scored in 1 HPF and in 10 consecutive HPFs (starting from the HPF with highest TB) and the absolute count of tumour buds was registered.

To evaluate minimal cell nest size (MCNS), the whole tumour was scanned for the cell nest consisting of the lowest number of tumour cells—cell nests were defined as clustered tumour cells or single invading tumour cells surrounded by stroma (in accordance with previous publications) [20, 31, 32]. The absolute number of tumour cells forming this smallest cell nest was documented with one single invading tumour cell defined as single-cell invasion (SCI). Therefore, in case of a budding tumour MCNS was per definition four or less whereas in cases without TB the MCNS comprised five or more tumour cells. Regarding all cases, MCNS ranged from one cell (SCI) up to 46 tumour cells.

For comparison with the previously described dissociation grading scheme based on TB combined with MCNS, each carcinoma was evaluated according as previously reported by assigning a sum score comprising TB (1–3 points) plus MCNS (1–4 points) yielding a three-tiered grading system: CDG-nG1; CDG-nG2; CDG-nG3 [20, 21, 30, 31].

Additionally, risk scores were assessed for all cases in accordance with the recommendations of Brandwein–Gensler et al. [25]. The risk score is composed of point assignments for perineural invasion (none: 0 points, nerves <1 mm diameter: 1 point, nerves ≥1 mm: 3 points), lymphocytic infiltrate at the invasive front (band-like infiltrate: 0 points, large patches: 1 point, mild or absent: 3 points) and the worst pattern of invasion at the invasive margin (broad or finger-like pushing border/tumour cell nests >15 cells: 0 points, tumour cell nests ≤15 cells: 1 point, tumour satellites ≥1 mm away from the main tumour mass: 3 points). All three point values were summed up with a sum score of 0 points representing low-risk cases, 1–2 points representing intermediate risk cases and 3–9 points representing high-risk cases. Only cases where the invasive margin of the tumour was displayed on the digitised slides could be analysed for the risk score (*n* = 307) as otherwise the worst pattern of invasion could not be determined.

### Cutpoints for TB and MCNS

**Fig. 2:**
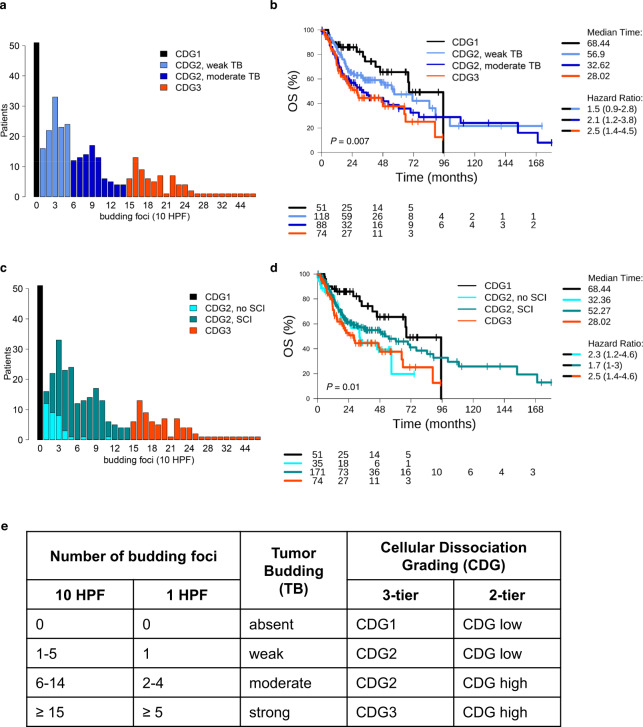
Optimisation of the cellular dissociation grading (CDG) system. Two four-tiered grading systems were investigated, both based on tumour classification as CDG1, CDG2 and CDG3 and an additional split of the CDG2 class. **a**, **b** The set of CDG2 tumours was split based on the number of budding foci. Hazard ratios compared to CDG1 tumours increased and reached 1.5, 2.1, and 2.5 for CDG2/weak TB, CDG2/moderate TB and CDG3 tumours. **c**, **d** The set of CDG2 tumours was split based on the presence or absence of single-cell invasion (SCI). Counterintuitively, CDG2 tumours without SCI showed a (non-significant) trend to unfavourable prognosis compared to CDG2 tumours with SCI. **e** Relation between the number of budding foci, the classification of TB, and the CDG systems.

Cutpoint optimisation was performed in the TB data generated using the 10-HPF method. In the study cohort, a multimodal distribution of TB was observed and cutpoints of 1, 6 and 15 tumour buds were chosen to separate the peaks in the distribution from each other (Fig. 2a). Additionally, the prognostic performance of different cutpoints for TB was analysed using the biostatistical tool “Cutoff Finder” [33], revealing 6 tumour buds as optimal cutpoint (Supplemental Fig. S1). Based on the determined TB cutpoints, a four-tiered, a three-tiered and a two-tiered tumour classification system were introduced. Concerning the four-tiered system, cutpoints separating absent, weak, moderate and strong TB were 0, 1–5, 6–14 and ≥15 TB, respectively. The 3-tiered system grouped absent vs. weak and moderate vs. strong TB and the two-tiered system found the optimum cutpoint to be six buds indicating a split between absent and weak vs. moderate and strong TB (Fig. 2b, e).

Analysing MCNS, 245 (74.0%) tumours showed TB with SCI, while 35 (10.6%) tumours showed TB without SCI and in the latter group 18 (5.4%), 13 (3.9%) and 4 (1.2%) had MCNS 2 tumour cells, 3 tumour cells and 4 tumour cells, respectively. Because the sample sizes of the subgroups with MCNS 2, 3, and 4 were not large enough for a statistically well-powered analysis, we concentrated the analysis of MCNS on SCI and its prognostic significance (Fig. 2c, d).

### Statistical analysis

Data analysis and visualisation were performed using the statistical language R [34]. Fisher’s exact test was used to determine differences between patient characteristics and categorised budding activity. Analysis of overall survival (OS) and progression-free interval (PFI) were performed using the Kaplan–Meier method and the log-rank test to assess the significance of differences in survival. Univariate and multivariate survival analyses were performed using the Cox proportional hazard models and Wald’s test to assess the significance of hazard ratios (HR). The relation between the 1-HPF and the 10-HPF method for determination of TB was analysed using linear regression with intercept set to zero. The Wilcoxon test and the Cochran–Armitage test were applied to test for increase of TB between ordered groups. All tests were conducted two-sided and *p*-values <0.05 were considered significant.

## Results

The study cohort included 286 HPV-positive and 45 HPV-negative HNSCC patients from TCGA (Supplemental Table S1).

### Evaluation of tumour budding (TB)

A four-tiered classification system was introduced in accord with the multimodal distribution of TB (Fig. 2a): While TB was absent in 15% of tumours, TB was weak (1–5 budding foci) in 36% of tumours, moderate (6–14 budding foci) in 27% of tumours and strong (≥15 budding foci) in 22% of tumours. Pooling cases with weak and moderate TB, we introduced a three-tiered cellular dissociation grading system (CDG1, CDG2, CDG3). Classification of the study cohort using the novel grading system coincided with classification using the established three-tiered grading system (CDG-nG1; CDG-nG2; CDG-nG3 [20]) that includes both tumour budding and MCNS. Higher grading with respect to the TB-based four-tiered classification system correlated with significantly shorter OS (Fig. 2b, *p* = 0.007). In particular, splitting according to TB resulted in a shorter overall survival (OS) of patients with TB moderate tumours (median survival: 33 months) compared to patients with TB weak tumours (median survival: 57 months).

### Evaluation of single-cell invasion (SCI)

In the study cohort, 245 (74%) samples showed TB with SCI and 35 (11%) samples showed TB without SCI. TB was absent in the remaining 51 (15%) samples. While tumours of CDG2 either did not have SCI or had SCI, all tumours of CDG3 had SCI (Fig. 2c). Splitting with respect to SCI resulted in a classification of 10% of the tumours as CDG2/SCI-negative and of 52% of the tumours as CDG2/SCI+ positive. Counterintuitively, the former tumours showed a non-significant trend to shorter OS compared to the latter tumours (median survival: 32 months vs. 52 months; Fig. 2d). Thus, splitting of the CDG2 subgroup by the level of TB (moderate vs. weak) allowed the extraction of additional prognostic information, while splitting with respect to MCNS (SCI vs. no SCI) did not.

### Updated cellular dissociation grading (CDG) systems

As the inclusion of SCI did not add additional prognostic information, two novel CDG systems based solely on TB (a three-tiered and a two-tiered system) were utilised and analysed throughout this study (Fig. 2e). A cutpoint of six tumour buds resulted in the most significant prognostic separation for both OS and PFI (Supplemental Fig. S1). This optimised cutpoint was one of the cut-offs identified by analysis of the multimodal TB distribution and the “Cutoff Finder” and was consequently included in the two-tiered CDG system to separate CDG high from CDG low tumours.

### Association of TB with clinicopathologic characteristics

TB was significantly higher in HPV-negative tumours, in conventional compared to basaloid tumours, in tumours with higher Brandwein–Gensler (BG) score, and in N + tumours (Table 1). TB was absent in 36% of the HPV-positive tumours, but only in 12% of the HPV-negative tumours (*p* < 0.001). While TB was absent in 33% of the basaloid tumours, it was absent in only 14% of the conventional tumours (*p* = 0.017). While TB was absent in 19% of the N0 tumours, it was absent in only 11% of N + tumours (*p* = 0.09). Also, TB was significantly different for different tumour localisations (*p* < 0.001). While TB was absent in 33% of oropharyngeal tumours, it was absent in only 20% of laryngeal tumours and only 11% of tumours of the oral cavity and lips, reflecting HPV-association. Correlation of TB with BG score and with nodal status remained significant when restricting the analysis to the subgroup of HPV-negative tumours (*n* = 286, Supplemental Table S2). No significant correlations of TB with clinicopathological tumour characteristics were detected in the subgroup of HPV-positive tumours, most probably due to the limited number of samples (*n* = 45, Supplemental Table S3).

**Table 1:** Association of tumor budding (TB) with clinicopathologic tumor characteristics in the TCGA-HNSC cohort.

Parameter	TB absent	TB weak	TB moderate	TB strong	*P* value
Age					0.92
≤61	25 (14.2%)	63 (35.8%)	47 (26.7%)	41 (23.3%)	
>61	26 (16.8%)	55 (35.5%)	41 (26.5%)	33 (21.3%)	
Sex					0.31
Female	13 (15.3%)	24 (28.2%)	28 (32.9%)	20 (23.5%)	
Male	38 (15.4%)	94 (38.2%)	60 (24.4%)	54 (22%)	
HPV					**<0.001**
Negative	35 (12.2%)	99 (34.6%)	84 (29.4%)	68 (23.8%)	
Positive	16 (35.6%)	19 (42.2%)	4 (8.9%)	6 (13.3%)	
Smoking					0.34
Non-smoker	15 (13.6%)	43 (39.1%)	33 (30%)	19 (17.3%)	
Smoker	34 (16.3%)	70 (33.5%)	53 (25.4%)	52 (24.9%)	
NA	2	5	2	3	
AJCC stage					0.51
Stage I	2 (13.3%)	3 (20%)	5 (33.3%)	5 (33.3%)	
Stage II	8 (16.7%)	19 (39.6%)	15 (31.2%)	6 (12.5%)	
Stage III	8 (16.7%)	20 (41.7%)	10 (20.8%)	10 (20.8%)	
Stage IV	25 (13%)	68 (35.2%)	49 (25.4%)	51 (26.4%)	
NA	8	8	9	2	
WHO grade					0.16
G1	10 (21.7%)	16 (34.8%)	12 (26.1%)	8 (17.4%)	
G2	24 (11.8%)	78 (38.2%)	59 (28.9%)	43 (21.1%)	
G3	17 (21%)	24 (29.6%)	17 (21%)	23 (28.4%)	
WHO subtype					**0.03**
Basaloid	8 (33.3%)	10 (41.7%)	4 (16.7%)	2 (8.3%)	
Conventional	42 (13.8%)	107 (35.1%)	84 (27.5%)	72 (23.6%)	
Papillary	0 (0%)	1 (100%)	0 (0%)	0 (0%)	
Verrucous	1 (100%)	0 (0%)	0 (0%)	0 (0%)	
Brandwein–Gensler score					**<0.001**
Low	8 (66.7%)	4 (33.3%)	0 (0%)	0 (0%)	
Intermediate	31 (16%)	72 (37.1%)	56 (28.9%)	35 (18%)	
High	8 (7.9%)	31 (30.7%)	25 (24.8%)	37 (36.6%)	
NA	4	11	7	2	
pN					**0.008**
N0	23 (19.2%)	50 (41.7%)	29 (24.2%)	18 (15%)	
N1/2/3	19 (11.4%)	51 (30.7%)	48 (28.9%)	48 (28.9%)	
NA	9	17	11	8	
pT					0.11
T1/T2	20 (17.4%)	33 (28.7%)	37 (32.2%)	25 (21.7%)	
T3/T4	24 (12.3%)	77 (39.5%)	46 (23.6%)	48 (24.6%)	
NA	7	8	5	1	
cpM					0.6
M0	49 (15.3%)	115 (35.9%)	84 (26.2%)	72 (22.5%)	
M1	1 (33.3%)	1 (33.3%)	0 (0%)	1 (33.3%)	
NA	1	2	4	1	
Localization					**<0.001**
Hypopharynx	0 (0%)	0 (0%)	1 (16.7%)	5 (83.3%)	
Larynx	17 (19.5%)	27 (31%)	27 (31%)	16 (18.4%)	
Oral cavity and lips	22 (10.9%)	75 (37.1%)	57 (28.2%)	48 (23.8%)	
Oropharynx	12 (33.3%)	16 (44.4%)	3 (8.3%)	5 (13.9%)	
L1					0.21
Absent	51 (15.8%)	112 (34.7%)	87 (26.9%)	73 (22.6%)	
Present	0 (0%)	6 (75%)	1 (12.5%)	1 (12.5%)	
Pn1					0.14
Absent	46 (17.4%)	95 (36%)	68 (25.8%)	55 (20.8%)	
Present	5 (7.5%)	23 (34.3%)	20 (29.9%)	19 (28.4%)	
Margin status					0.67
Negative/close	38 (14.4%)	87 (33.1%)	75 (28.5%)	63 (24%)	
Positive	6 (14%)	18 (41.9%)	9 (20.9%)	10 (23.3%)	
NA	7	13	4	1	

Significant *P* values are shown in bold.

### Prognostic impact of CDG

**Fig. 3:**
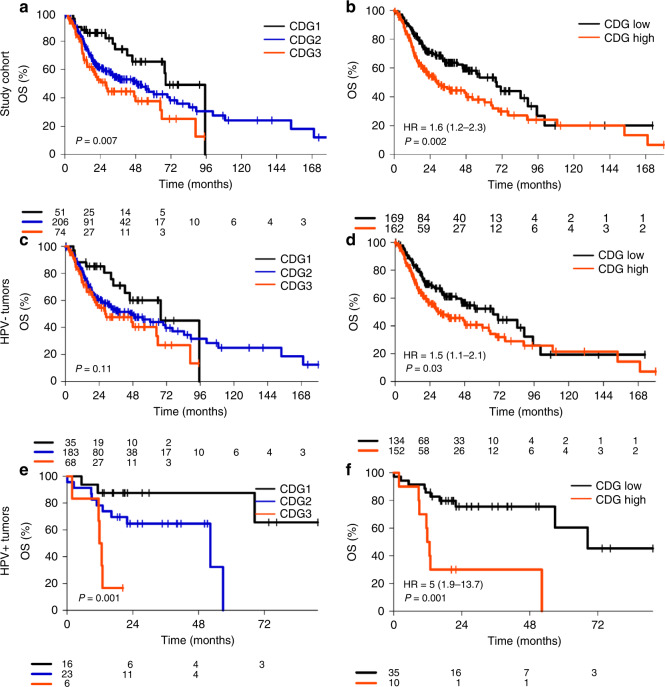
Association of overall survival (OS) with cellular dissociation grading (CDG). Performance of the 3-tiered CDG system and of the 2-tiered CDG system in the study cohort (**a, b**), in the subcohort of HPV-negative tumours (**c, d**) and in the subcohort of HPV-positive tumours (**e, f**).

We analysed the prognostic significance of the three-tiered and two-tiered CDG systems (Fig. 3, Supplemental Fig. S2). The three-tiered CDG system was significantly associated with altered OS and PFI in the whole study cohort (*p* = 0.007 and *p* = 0.05) and in HPV-positive tumours (*p* = 0.001 and *p* = 0.002), whereas there was no significant correlation of grading in the HPV-negative subgroup (*p* = 0.11 and *p* = 0.37). The two-tiered grading was associated with significantly altered OS in the study cohort (HR = 1.64, *p* = 0.002), in HPV-negative tumours (HR = 1.48, *p* = 0.03), and in HPV-positive tumours (HR = 5.05, *p* = 0.001). Furthermore, the two-tiered grading system was associated with altered PFI in the study cohort and in HPV-positive tumours, but not in HPV-negative tumours. In summary, the two-tiered CDG system outperformed the three-tiered CDG system in terms of significance in all analysed subgroups and for both endpoints.

### Subgroup analysis

**Fig. 4:**
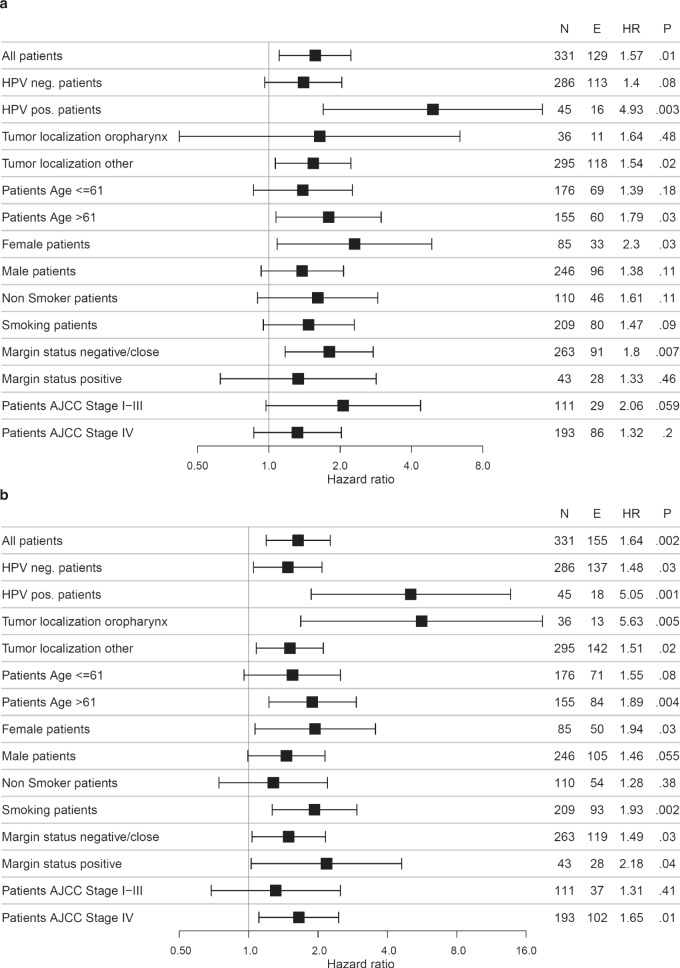
Subgroup analysis of the prognosticity of cellular dissociation grading (CDG). Comparison of CDG high with CGD low tumours with respect to PFI (**a**) and OS (**b**). N, number of patients, E, number of events, HR, hazard ratio, CI, 95% confidence interval.

Subgroup analyses (Fig. 4, Supplementary Fig. S3) revealed a stronger impact of CDG on OS in HPV-positive tumours (HR = 5.05, *p* = 0.001) compared to HPV-negative tumours (HR = 1.48, *p* = 0.03), in oropharyngeal tumours (HR = 5.63, *p* = 0.005) compared to tumours at other localisations (HR = 1.51, *p* = 0.02), for smokers (HR = 1.93, *p* = 0.002) compared to non-smokers (HR = 1.28, *p* = 0.38), as well as in stage III and stage IV tumours (HR = 1.68, *p* = 0.29 and HR = 1.65, *p* = 0.01) compared to stage II tumours (HR = 1.11, *p* = 0.83). By contrast, the impact of CDG on OS did not vary much with patient age, sex and tumour margin status. As for OS, the impact of CDG on PFI was stronger in HPV-positive compared to HPV-negative tumours (HR = 4.93, *p* = 0.003 vs. HR = 1.54, *p* = 0.02). In contrast to OS, the impact of CDG on PFI was stronger in stage II tumours (HR = 2.73, *p* = 0.1) compared to stage III and stage IV tumours (HR = 1.19, *p* = 0.76 and HR = 1.32, *p* = 0.2). In stage I tumours, the number of events for both OS and PFI was too small for statistical analysis.

### Comparison of cellular dissociation grading, Brandwein–Gensler risk model and WHO grading

The prognostic power of the three grading systems was evaluated in univariate and multivariate analyses of PFI and OS (Table 2, Supplemental Table S4–S7). We performed a multivariate analysis separately for each of the three grading systems (including age, sex, HPV status, localisation, AJCC stage, tumour margin) and an additional multivariate analysis including all three gradings. To this end, tumours with low and intermediate BG scores were pooled, because of the very small number of BG-low tumours in the study cohort (Supplemental Fig. S4). WHO G2 and G3 tumours were pooled, because of a twist of Kaplan–Meier curves with G2 tumours showing numerical shorter survival than G3 tumours (Supplemental Fig. S5). These poolings resulted in three two-tiered grading systems that could be directly compared.

**Table 2:** Prognostic performance of cellular dissociation grading (CGD), Brandwein–Gensler (BG) histopathological risk score, and WHO grading in the TCGA-HNSC cohort (*n* = 311) and in the subcohorts of HPV-negative (*n* = 286) and HPV-positive (*n* = 45) tumors.

Outcome	CDG (high vs. low)			BG score (high vs. int./low)			WHO grade (G3/G2 vs. G1)		
	HR	CI	*P*	HR	CI	*P*	HR	CI	*P*
*Univariate analysis*									
PFI	**1.57**	**1.1–2.22**	**0.012**	**1.51**	**1.04–2.18**	**0.029**	1.64	0.92–2.91	0.092
OS	**1.64**	**1.19–2.26**	**0.0024**	**1.53**	**1.1–2.13**	**0.012**	**1.69**	**1.01–2.81**	**0.046**
HPV−: PFI	1.4	0.96–2.03	0.08	1.42	0.96–2.12	0.081	1.63	0.91–2.92	0.097
HPV−: OS	**1.48**	**1.05–2.08**	**0.025**	1.38	0.96–1.97	0.079	**1.69**	**1.01–2.83**	**0.047**
HPV+: PFI	**4.93**	**1.7–14.3**	**0.0034**	2.71	0.87–8.42	0.084	NA^a^	NA^a^	NA^a^
HPV+: OS	**5.05**	**1.87–13.67**	**0.0014**	**3.95**	**1.37–11.44**	**0.011**	NA^a^	NA^a^	NA^a^
*Multivariate analysis*									
PFI	**1.55**	**1.05–2.28**	**0.028**	1.49	1–2.23	0.051	1.18	0.64–2.19	0.6
OS	**1.74**	**1.21–2.49**	**0.0026**	**1.48**	**1.03–2.13**	**0.033**	1.43	0.81–2.52	0.22
HPV−: PFI	1.37	0.92–2.06	0.12	1.3	0.84–2.01	0.24	1.1	0.59–2.06	0.76
HPV−: OS	**1.51**	**1.04–2.18**	**0.03**	1.24	0.83–1.84	0.29	1.24	0.7–2.21	0.46
HPV+: PFI	3.46	0.82–14.56	0.09	2.07	0.46–9.37	0.34	NA^a^	NA^a^	NA^a^
HPV+: OS	2.28	0.73–7.17	0.16	2.63	0.71–9.69	0.15	NA^a^	NA^a^	NA^a^

Univariate analysis of DFI and OS and multivariate analysis of DFI and OS including age (>61 vs. ≤61 years), sex (female, male), HPV status (HPV-positive, HPV-negative), localization (oropharynx, all other), AJCC stage (I–III, IV), and margin status (negative/close, positive).

Bold fonts indicate statistical significance.

*HR* hazard ratio, *CI* 95% confidence interval.

^a^Analysis not feasible, because of low sample size (only a single tumor was HPV+ and G1).

High CDG was associated with significantly shorter PFI and OS in the study cohort in univariate and in multivariate analysis. High BG scores were associated with significantly shorter PFI and OS in univariate analysis and with significantly shorter OS but not PFI in multivariate analysis. High WHO grade was associated with significantly shorter OS in univariate analysis, but not in multivariate analysis and not with significantly shorter PFI. In the subgroup of HPV-negative tumours, CDG was a significant prognostic marker for OS in univariate and in multivariate analysis, WHO grade was a significant prognostic marker only in univariate analysis, while the BG score did not reach significance. In the subgroup of HPV-positive tumours, CDG and BG score were significant prognostic markers for OS in univariate analysis, while analysis of WHO grading was not feasible due to a small number of samples.

In a multivariate analysis of OS including all three grading systems and clinicopathological tumour characteristics, CDG remained as significant prognostic factor (HR = 1.53, CI 1.06–2.21, *p* = 0.02), while the BG score and WHO grading did not reach significance (Supplemental Table S7). In summary, CDG outperformed the two other grading systems in a prognostic stratification of the study cohort, the subcohort of HPV-negative tumours, and the subcohort of HPV-positive tumours.

### Evaluation of TB in a single HPF

**Fig. 5:**
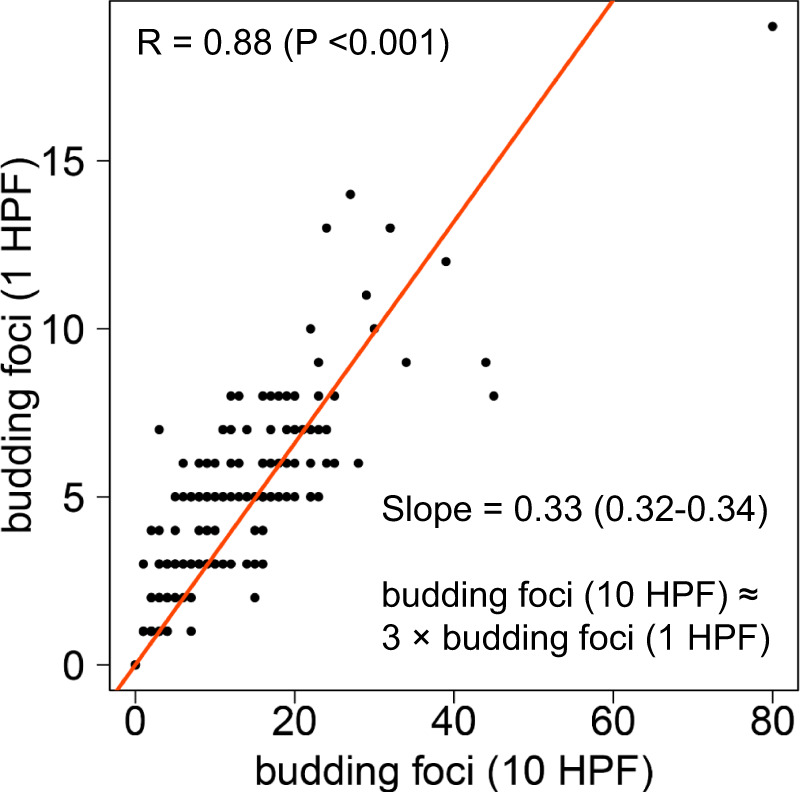
Comparison of the 1-HPF and the 10-HPF method for the evaluation of TB. Evaluation of TB in 10 high-power fields (HPF) and in a single HPF. Linear regression revealed that the detected number of budding foci differed by a factor of about three.

To translate the evaluation of TB to small tissue biopsies, we analysed TB in a single HPF simulating a situation in which only a limited tissue area is available. The numbers of budding foci detected in 1 and in 10 HPF were strongly correlated (R = 0.88, *p* < 0.001, Fig. 5). Linear regression revealed that the number of buds detected by the 10-HPF method were about threefold higher than the number of buds detected by the 1-HPF method. Based on that, we converted TB cutpoints for the 10-HPF method to TB cutpoints for the 1-HPF method (Fig. 2e). As the study cohort included only resection specimen which according to the International Tumor Budding Consensus Conference (ITBCC) allows to account for the heterogeneity in TB distribution along with higher interobserver agreement [16] most of our analyses were based on the 10-HPF approach.

### Detection of clinically occult lymph-node metastases using TB

**Fig. 6:**
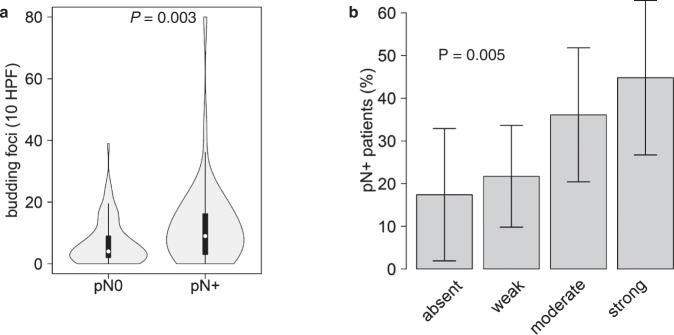
Analysis of clinically nodal-negative (cN0) patients. **A** Higher numbers of budding foci in pathologically nodal-positive (pN+) compared to pN0 patients. **B** Increasing percentages of pN+ patients with increasing TB.

Nodal-positive patients with clinically negative lymph-node status (cN0/pN+) showed significantly higher TB compared to patients with clinicallly negative nodes which were confirmed negative inpathologic evaluation (cN0/pN0) with a median of 9 (CDG high) vs. 4 buds (CDG low) in 10 HPF (*p* = 0.003, Fig. 6). The percentage of pN+ patients gradually increased and reached 17%, 22%, 36%, and 45% in TB absent, TB weak, TB moderate and TB strong cN0 HNSCC (*p* = 0.005). Using the 1-HPF approach, we obtained a similar result with a median of 5 buds vs. 3 buds (*p* = 0.006) and percentages reaching 17% (TB absent), 17% (TB weak), 25% (TB moderate), and 45% (TB strong; *p* = 0.003, Supplemental Fig. S6). These results support the notion that analysis of TB could support the detection of clinically occult lymph-node metastasis and that TB grading can be conducted in small (pre-operative) biopsies by using the 1-HPF method.

## Discussion

For HNSCC patients established parameters such as the WHO histological grade do not provide sufficient prognostic or predictive information for personalised therapies. Indeed, the current WHO classification itself states that “conventional” histological grading for HPV-negative HNSCC (based on Broder´s grading system proposed in the 1920´s [29]) does not provide any prognostic information [11]. For HPV-positive HNSCC, grading is not even recommended anymore [35–37].

To contribute to the development of an improved histopathological grading system, we analysed the prognostic significance of TB and MCNS and optimised the up to date arbitrarily determined cutpoints in the TCGA HNSCC cohort. We identified a cutpoint of 6 buds (for the 10-HPF method) which allowed stratification into CDG low and CDG-high subgroups in HPV-negative and—for the first time—HPV-positive HNSCC. CDG low and CDG-high subgroups differed significantly concerning OS and PFI in univariate and multivariate analyses emphasising the relevance of TB as independent biomarker for adverse clinical outcomes.

Therefore, the evaluation of TB could add further prognostic significance to conventional risk scoring models based on established clinicopathologic parameters like age or AJCC stage and help to guide treatment decisions [38, 39]. Furthermore, as CDG is applicable and prognostic in 1 HPF (a tissue size which might simulate a biopsy specimen), we assume that CDG is feasible as well in cases in which limited cancer tissue is available for evaluation.

The histomorphologic pattern MCNS did not provide additional prognostic information in combination with TB. In line with this finding, the application of the previously proposed grading scheme [20, 21] consisting of TB and MCNS did not yield statistically robust results in the TCGA cohort. Furthermore, the former grading scheme was not able to stratify patients of the HPV-negative or -positive TCGA HNSCC subgroup according to their prognosis.

Considering our results in the context of previous studies analysing TB in HNSCC, which up to date included exclusively HPV-negative cancers, the prognostic impact of TB on various HNSCC locations with HPV-independent carcinogenesis was confirmed [20–22, 40–42].

Although generally being associated with improved survival, HPV-positive HNSCC represents a heterogeneous subgroup of HNSCC with poor survival rates in 25% of HPV-positive cases [26, 43, 44]. Therefore, our proposed grading system might aid to identify low-risk patients (CDG low) with HPV-positive HNSCC who might benefit from treatment de-escalation, but also high-risk patients (CDG high) who could benefit from intensified treatment regimens. Stating this, it has to be considered that the cohort size of HPV-positive tumours is comparably small, limiting the robustness of our results for CDG in this subgroup. Yet, to the best of our knowledge this is the first study reporting data on TB in HPV-positive tumours and we believe that a further prognostic stratification of HPV-positive patients is indispensable in this rather heterogeneous subset of patients [26, 43, 44]. Therefore, the prognostic significance of CDG in HPV-positive tumours should be validated in future studies aiming at stable identification of high-risk HPV-positive tumours.

Comparing the proposed TB-based CDG model with established risk stratification systems like WHO grading [11, 29] and the BG risk model [25] the CDG system outperformed both other models in univariate and multivariate analyses, respectively. CDG was the only grading which was independently prognostic for both OS and PFI, while BG reached independent prognostic significance only for OS and the WHO grading showed no independent prognostic impact at all. In a mutivariate analysis including all three gradings, CDG remained the only signficant prognostic factor while the other two gradings were dropping out. Furthermore, while CDG is applicable to all cases, analysis of BG is hampered by the fact that the invasive front has to be evaluated [25]—a tumour region which is not covered by the cancer specimens in all clinical cases. Therefore, we propose the determination of CDG in clinical practice due to its high and independent prognostic relevance. Taking into account the impact of BG especially on OS, we could imagine that CDG and the BG risk model might complement each other, increasing the prognostic relevance of a combined model. However, so far there are no studies combining TB and the BG risk model regarding their mutual prognostic relevance warrenting future studies tailored to this question.

Our results are in line with previous literature comparing grading systems in HNSCC [37, 45–47]. WHO grading, which is based on tumour differentiation features (e.g. cellular pleomorphism) proposed by Broders in the 1920s [29] failed to prove its prognostic impact in several studies and its limited prognostic value is widely accepted [35, 45, 48]. Evaluation of BG showed inconsistent results concerning its prognostic significance [45, 49, 50]. The limitation of this model is extensively reviewed by Sawazaki-Calone and Rodrigues (and goes beyond our article) [37, 51]. Depth of invasion has been shown to be a prognostic parameter especially in oral HNSCC and was included in the “Budding and depth of invasion” model by Almangush et al. [37, 45, 52]. However, depth of invasion is rather a staging than a grading parameter [20]. Moreover, in our cohort, it could not be determined in many cases as (1) only one diagnostic slide per case was available and we could not reconstruct if it was the one showing the deepest invasion, and (2) several slides were unoriented rendering the measurement of depth of invasion impossible.

The optimal treatment of patients with clinically negative cervical lymph nodes is currently subject of debate as a relatively high percentage of clinically node negative patients (20–30%) show lymph-node metastases after histologic workup [53, 54]. We demonstrated the association of TB with lymph-node metastases in clinically negative necks and an increasing probability of occult lymph-node metastases with higher TB both for the assessment of TB in 1 and in 10 HPF. Thus, TB assessment may represent an easy to conduct and cost-effective method to identify patients with an elevated risk of occult lymph-node metastases who should undergo elective neck dissection [23]. The evaluation of 1 HPF takes into account a small tissue area. This may simulate a biopsy situation, in which only a small cancer specimen is feasible for analysis. Therefore, the association of TB in 1 HPF and lymph-node metastases apears to be transferable to an excisional biopsy sample and we assume that our proposed grading could aid in the treatment planning of neck dissections in future. Nevertheless, future studies are required explicitly investigating the association of CDG and lymph-node metastases in a biopsy setting.

While it could be shown that TB evaluation based on immunohistochemical stainings (pancytokeratin) is more sensitive compared to the evaluation on H&E-stained slides, the utilisation of immunohistochemical stainings does not increase the prognostic significance of TB [55]. Indeed, it was demonstrated that TB is overestimated by immunohistochemical stainings and that different cut-offs in this setting might be required [55, 56]. Hence, we believe that TB should be analysed on H&E-stained slides due to standardisation purposes and due to its relatively easy applicability and cost-effectiveness.

There are a few limitations of the current study. The proposed 2-tiered CDG scheme differs from the three-tiered system recommended by the ITBCC for colorectal cancer [16]. Nevertheless, we believe that the utilisation of our two-tiered CDG scheme is legitimate as cut-offs suggested by the ITBCC support decision making in ambiguous pT1 colorectal cancer and stage II colorectal cases but are not optimised for other tumour entities—in particular as colorectal cancer represents an adenocarcinoma whereas HNSCC are of squamous cell origin [57, 58]. Therefore, this scheme might not be optimised for grading of HNSCC [16]. Furthermore, the proposed cutoff (6 buds per 10 HPF) differs from cutpoints applied in previous studies hindering the direct comparability of results [59–61]. Nevertheless, our statistical approach aimed to optimise cutoff points with two established statistical methods and thus we think the applicability and prognostic relevance of our proposed cutoff points should be tested in future studies.

Due to the small number of AJCC stage I and II tumours the subgroup of early stage disease was underpowered for the analysis of the prognostic significance of CDG. However, previous publications have reported the prognostic relevance of TB in this patient subset [9, 62, 63].

The study cohort included conventional, basaloid, verrucous, and papillary HNSCC, while sarcomatoid tumours were excluded for the following reasons: (1) There were only *n* = 2 cases with sarcomatoid histology in the TCGA cohort hampering statistical evaluation, and (2) to the best of our knowledge there are no published studies on the evaluation of TB in sarcomatoid HNSCC.

The CDG system introduced in this study needs to be confirmed in a further retrospective and ideally prospective studies. While TB is a well-established prognostic marker in HPV-negative HNSCC, the new CDG system including cutpoints needs to be reconfirmed. For HPV-negative HNSCC, the prognosticity of TB in general and the new CDG system need to be reconfirmed. The validations should include cases with the entire histology material available to ensure control of the intra-block variability.

Taken together, this is the first study establishing a prognostic value of TB in HPV-positive HNSCC, a finding that should be validated in further study cohorts. As an ultimate goal, TB should be developed further as a tool for therapy guidance in HPV-positive and -negative HNSCC requiring additional steps of retrospective and prospective validation ideally in the setting of clinical trials with treatment-naive patients and potentially as well in clinical trials probing targeted therapy approaches or immunotherapy.

## Conclusions

TB can be assessed easily and cost effectively in clinical practice based on HE-stained (digitised) slides. The systematic analysis of TB cutpoints yielded new and optimised values with improved independent prognostic significance in HPV-negative and HPV-positive HNSCC. The evaluation of MCNS did not provide additional prognostic relevance. The TB-based CDG grading scheme was shown to be an independent prognostic parameter yielding superior prognostic significance compared to established grading systems like WHO grading or the BG risk model. Our study is the first to show that TB is able to stratify patients with HPV-positive HNSCC into low-grade and high-grade subgroups. CDG-high cases are more frequently associated with occult lymph-node metastases and inferior clinical outcomes which might help to identify HNSCC patients who could benefit from more radical treatment approaches. The suitability of TB for therapy prediction should be addressed in future studies.

### Supplementary information

Supplemental Tables and Figures

STROBE checklist

## Data Availability

The datasets generated and/or analysed during the current study are available from the corresponding author on reasonable request.
