# Correction: Tumour budding-based grading as independent prognostic biomarker in HPV-positive and HPV-negative head and neck cancer

**DOI:** 10.1038/s41416-023-02521-6

**Published:** 2024-01-08

**Authors:** Fabian Stögbauer, Susanne Beck, Iordanis Ourailidis, Jochen Hess, Christopher Poremba, Maren Lauterbach, Barbara Wollenberg, Anna Maria Stefanie Buchberger, Moritz Jesinghaus, Peter Schirmacher, Albrecht Stenzinger, Wilko Weichert, Melanie Boxberg, Jan Budczies

**Affiliations:** 1 Institute of Pathology, School of Medicine, Technical University of Munich (TUM), 81675 Munich, Germany; 2 University of Heidelberg, Institute of Pathology, Im Neuenheimer Feld 224, 69120 Heidelberg, Germany; 3 Section Experimental and Translational Head and Neck Oncology, Department of Otolaryngology, Head and Neck Surgery, University Heidelberg, Im Neuenheimer Feld 400, 69120 Heidelberg, Germany; 4 Research Group Molecular Mechanisms of Head and Neck Tumors, German Cancer Research Center (DKFZ), Im Neuenheimer Feld 280, 69120 Heidelberg, Germany; 5 Pathologie München-Nord, 80992 Munich, Germany; 6 Department of Otorhinolaryngology Head and Neck Surgery, University Hospital Klinikum Rechts der Isar, Ismaningerstr. 22, 81675 Munich, Germany; 7 Institute of Pathology, University Hospital Marburg, Baldingerstraße, 35043 Marburg, Germany; 8 German Cancer Consortium (DKTK), Munich and Heidelberg Partner Sites, Munich and Heidelberg, Germany

**Keywords:** Prognostic markers, Surgical oncology, Prognostic markers

Correction to: *British Journal of Cancer* 10.1038/s41416-023-02240-y, published online 12 April 2023

The original version of this article contained a mistake. In the Methods section, under the heading TCGA cohort, the sentence originally read as follows:

“HPV status was determined based on the number of RNA sequencing reads aligning to the viral genes E6 and E7 as previously described [27, 28].”

The corrected version is:

“HPV status was determined by investigating the tumour DNA with a PCR based assay interrogating 16 HPV types (16, 18, 31, 33, 35, 39, 45, 51, 52, 56, 58, 59, 66, 68, 73, and 90) as previously described [27]. Tumours were classified as HPV+ when the test result was positive, as HPV- when the test result was negative and excluded from the analysis when the test result was indeterminate.”

The complete, corrected section can be found below. The correction does not have any effect on the results or conclusions of the paper.

TCGA cohort

The TCGA-HNSC cohort included a total of 528 patients who were treated for HNSCC [27, 28]. Digitised H&E-stained diagnostic slides of 471 cases were available from the GDC Data Portal (https://portal.gdc.cancer.gov). A total of 331 tumours consisting of conventional, basaloid, verrucous, and papillary HNSCC were included into the study after exclusion of 140 cases. Cases were excluded due to the following reasons: small biopsy specimen precluding the analysis of 10 HPF, a different tumour entity, sarcomatoid histomorphology, not enough tumour on slide, inferior scan quality, exposed carcinoma without relation to surrounding stroma precluding the analysis of TB, duplicates and a history of neoadjuvant treatment. HPV status was determined by investigating the tumour DNA with a PCR based assay interrogating 16 HPV types (16, 18, 31, 33, 35, 39, 45, 51, 52, 56, 58, 59, 66, 68, 73, and 90) as previously described [27]. Tumours were classified as HPV+ when the test result was positive, as HPV- when the test result was negative and excluded from the analysis when the test result was indeterminate. The clinicopathological characteristics of the study cohort are shown in Supplementary Table S1.

The original article has been corrected.

### Supplementary information

Supplementary Table S1

