# Supplementary material for: Tumour budding-based grading as independent prognostic biomarker in HPV-positive and HPV-negative head and neck cancer
Source: Br J Cancer. 2023 Apr 12;128(12):2295–306. doi: 10.1038/s41416-023-02240-y (PMC10241901; doi:10.1038/s41416-023-02240-y)
Supplement: Supplementary file 1 — Supplemental Tables and Figures [file 41416_2023_2240_MOESM1_ESM.pdf]

Supplemental Table S1: Clinicopathologic characteristics of the TCGA-HNSC cohort.

**Parameter**	**all patients**	**HPV-negative**	**HPV-positive**	**P-value**
**Total**	331	286	45	
**Age**				**.004**
**<=61**	176 (53.2%)	143 (81.2%)	33 (18.8%)	
**>61**	155 (46.8%)	143 (92.3%)	12 (7.7%)	
**Gender**				**.04**
**Female**	85 (25.7%)	79 (92.9%)	6 (7.1%)	
**Male**	246 (74.3%)	207 (84.1%)	39 (15.9%)	
**Smoking**				.87
**Non-Smoker**	110 (34.5%)	94 (85.5%)	16 (14.5%)	
**Smoker**	209 (65.5%)	180 (86.1%)	29 (13.9%)	
**NA**	12	12	0	
**AJCC Stage**				.51
**Stage I**	15 (4.9%)	15 (100%)	0 (0%)	
**Stage II**	48 (15.8%)	41 (85.4%)	7 (14.6%)	
**Stage III**	48 (15.8%)	41 (85.4%)	7 (14.6%)	
**Stage IV**	193 (63.5%)	169 (87.6%)	24 (12.4%)	
**NA**	27	20	7	
**WHO Grade**				**.002**
**G1**	46 (13.9%)	45 (97.8%)	1 (2.2%)	
**G2**	204 (61.6%)	179 (87.7%)	25 (12.3%)	
**G3**	81 (24.5%)	62 (76.5%)	19 (23.5%)	
**WHO Subtype**				**.005**
**basaloid**	24 (7.3%)	15 (62.5%)	9 (37.5%)	
**conventional**	305 (92.1%)	269 (88.2%)	36 (11.8%)	
**papillary**	1 (0.3%)	1 (100%)	0 (0%)	
**verrucous**	1 (0.3%)	1 (100%)	0 (0%)	
**Brandwein-Gensler score**				.17
**low**	12 (3.9%)	9 (75%)	3 (25%)	
**intermediate**	194 (63.2%)	173 (89.2%)	21 (10.8%)	
**high**	101 (32.9%)	85 (84.2%)	16 (15.8%)	
**NA**	24	19	5	
**pN**				**.03**
**N0**	120 (42%)	112 (93.3%)	8 (6.7%)	
**N1/2/3**	166 (58%)	140 (84.3%)	26 (15.7%)	
**NA**	45	34	11	
**pT**				**.007**
**T1/T2**	115 (37.1%)	93 (80.9%)	22 (19.1%)	
**T3/T4**	195 (62.9%)	179 (91.8%)	16 (8.2%)	
**NA**	21	14	7	
**cpM**				.35
**M0**	320 (99.1%)	278 (86.9%)	42 (13.1%)	
**M1**	3 (0.9%)	2 (66.7%)	1 (33.3%)	
**NA**	8	6	2	
**Localization**				**< .001**
**Hypopharynx**	6 (1.8%)	4 (66.7%)	2 (33.3%)	
**Larynx**	87 (26.3%)	84 (96.6%)	3 (3.4%)	
**Oral cavity and lips**	202 (61%)	186 (92.1%)	16 (7.9%)	
**Oropharynx**	36 (10.9%)	12 (33.3%)	24 (66.7%)	
**L1**				.08
**absent**	323 (97.6%)	281 (87%)	42 (13%)	
**present**	8 (2.4%)	5 (62.5%)	3 (37.5%)	
**Pn1**				.11
**absent**	264 (79.8%)	224 (84.8%)	40 (15.2%)	
**present**	67 (20.2%)	62 (92.5%)	5 (7.5%)	
**Margin status**				.2
**negative/close**	263 (85.9%)	234 (89%)	29 (11%)	
**positive**	43 (14.1%)	35 (81.4%)	8 (18.6%)	
**NA**	25	17	8	

Supplemental Table S2: Association of tumor budding (TB) with the clinicopathologic tumor characteristics in the HPV-negative subcohort of TCGA-HNSC.

Parameter	TB absent	TB weak	TB moderate	TB strong	P-value
**Age**					.66
**<=61**	15 (10.5%)	47 (32.9%)	45 (31.5%)	36 (25.2%)	
**>61**	20 (14%)	52 (36.4%)	39 (27.3%)	32 (22.4%)	
**Gender**					.36
**Female**	12 (15.2%)	22 (27.8%)	27 (34.2%)	18 (22.8%)	
**Male**	23 (11.1%)	77 (37.2%)	57 (27.5%)	50 (24.2%)	
**Smoking**					.52
**Non-Smoker**	10 (10.6%)	35 (37.2%)	31 (33%)	18 (19.1%)	
**Smoker**	23 (12.8%)	59 (32.8%)	51 (28.3%)	47 (26.1%)	
**NA**	2	5	2	3	
**AJCC Stage**					.39
**Stage I**	2 (13.3%)	3 (20%)	5 (33.3%)	5 (33.3%)	
**Stage II**	3 (7.3%)	18 (43.9%)	15 (36.6%)	5 (12.2%)	
**Stage III**	4 (9.8%)	18 (43.9%)	9 (22%)	10 (24.4%)	
**Stage IV**	21 (12.4%)	56 (33.1%)	46 (27.2%)	46 (27.2%)	
**NA**	5	4	9	2	
**WHO Grade**					.13
**G1**	10 (22.2%)	15 (33.3%)	12 (26.7%)	8 (17.8%)	
**G2**	17 (9.5%)	67 (37.4%)	56 (31.3%)	39 (21.8%)	
**G3**	8 (12.9%)	17 (27.4%)	16 (25.8%)	21 (33.9%)	
**WHO Subtype**					.41
**basaloid**	3 (20%)	6 (40%)	4 (26.7%)	2 (13.3%)	
**conventional**	31 (11.5%)	92 (34.2%)	80 (29.7%)	66 (24.5%)	
**papillary**	0 (0%)	1 (100%)	0 (0%)	0 (0%)	
**verrucous**	1 (100%)	0 (0%)	0 (0%)	0 (0%)	
**Brandwein-Gensler score**					**< .001**
**low**	5 (55.6%)	4 (44.4%)	0 (0%)	0 (0%)	
**intermediate**	22 (12.7%)	63 (36.4%)	55 (31.8%)	33 (19.1%)	
**high**	5 (5.9%)	24 (28.2%)	23 (27.1%)	33 (38.8%)	
**NA**	3	8	6	2	
**pN**					**.009**
**N0**	17 (15.2%)	48 (42.9%)	29 (25.9%)	18 (16.1%)	
**N1/2/3**	13 (9.3%)	40 (28.6%)	44 (31.4%)	43 (30.7%)	
**NA**	5	11	11	7	
**pT**					.19
**T1/T2**	11 (11.8%)	26 (28%)	34 (36.6%)	22 (23.7%)	
**T3/T4**	20 (11.2%)	69 (38.5%)	45 (25.1%)	45 (25.1%)	
**NA**	4	4	5	1	
**cpM**					.38
**M0**	34 (12.2%)	96 (34.5%)	81 (29.1%)	67 (24.1%)	
**M1**	1 (50%)	1 (50%)	0 (0%)	0 (0%)	
**NA**	0	2	3	1	
**Localization**					.36
**Hypopharynx**	0 (0%)	0 (0%)	1 (25%)	3 (75%)	
**Larynx**	15 (17.9%)	26 (31%)	27 (32.1%)	16 (19%)	
**Oral cavity and lips**	19 (10.2%)	68 (36.6%)	53 (28.5%)	46 (24.7%)	
**Oropharynx**	1 (8.3%)	5 (41.7%)	3 (25%)	3 (25%)	
**L1**					.8
**absent**	35 (12.5%)	96 (34.2%)	83 (29.5%)	67 (23.8%)	
**present**	0 (0%)	3 (60%)	1 (20%)	1 (20%)	
**Pn1**					.37
**absent**	31 (13.8%)	78 (34.8%)	65 (29%)	50 (22.3%)	
**present**	4 (6.5%)	21 (33.9%)	19 (30.6%)	18 (29%)	
**Margin status**					.8
**negative/close**	27 (11.5%)	78 (33.3%)	72 (30.8%)	57 (24.4%)	
**positive**	4 (11.4%)	13 (37.1%)	8 (22.9%)	10 (28.6%)	
**NA**	4	8	4	1	

Supplemental Table S3: Association of tumor budding (TB) with the clinicopathologic tumor characteristics in the HPV-positive subcohort of TCGA-HNSC.

Parameter	TB absent	TB weak	TB moderate	TB strong	P-value
**Age**					.3
**<=61**	10 (30.3%)	16 (48.5%)	2 (6.1%)	5 (15.2%)	
**>61**	6 (50%)	3 (25%)	2 (16.7%)	1 (8.3%)	
**Gender**					.25
**Female**	1 (16.7%)	2 (33.3%)	1 (16.7%)	2 (33.3%)	
**Male**	15 (38.5%)	17 (43.6%)	3 (7.7%)	4 (10.3%)	
**Smoking**					.66
**Non-Smoker**	5 (31.2%)	8 (50%)	2 (12.5%)	1 (6.2%)	
**Smoker**	11 (37.9%)	11 (37.9%)	2 (6.9%)	5 (17.2%)	
**AJCC Stage**					.09
**Stage I**	0 (0%)	0 (0%)	0 (0%)	0 (0%)	
**Stage II**	5 (71.4%)	1 (14.3%)	0 (0%)	1 (14.3%)	
**Stage III**	4 (57.1%)	2 (28.6%)	1 (14.3%)	0 (0%)	
**Stage IV**	4 (16.7%)	12 (50%)	3 (12.5%)	5 (20.8%)	
**NA**	3	4	0	0	
**WHO Grade**					.76
**G1**	0 (0%)	1 (100%)	0 (0%)	0 (0%)	
**G2**	7 (28%)	11 (44%)	3 (12%)	4 (16%)	
**G3**	9 (47.4%)	7 (36.8%)	1 (5.3%)	2 (10.5%)	
**WHO Subtype**					.41
**basaloid**	5 (55.6%)	4 (44.4%)	0 (0%)	0 (0%)	
**conventional**	11 (30.6%)	15 (41.7%)	4 (11.1%)	6 (16.7%)	
**papillary**	0 (0%)	0 (0%)	0 (0%)	0 (0%)	
**verrucous**	0 (0%)	0 (0%)	0 (0%)	0 (0%)	
**Brandwein-Gensler score**					.19
**low**	3 (100%)	0 (0%)	0 (0%)	0 (0%)	
**intermediate**	9 (42.9%)	9 (42.9%)	1 (4.8%)	2 (9.5%)	
**high**	3 (18.8%)	7 (43.8%)	2 (12.5%)	4 (25%)	
**NA**	1	3	1	0	
**pN**					.08
**N0**	6 (75%)	2 (25%)	0 (0%)	0 (0%)	
**N1/2/3**	6 (23.1%)	11 (42.3%)	4 (15.4%)	5 (19.2%)	
**NA**	4	6	0	1	
**pT**					.6
**T1/T2**	9 (40.9%)	7 (31.8%)	3 (13.6%)	3 (13.6%)	
**T3/T4**	4 (25%)	8 (50%)	1 (6.2%)	3 (18.8%)	
**NA**	3	4	0	0	
**cpM**					.21
**M0**	15 (35.7%)	19 (45.2%)	3 (7.1%)	5 (11.9%)	
**M1**	0 (0%)	0 (0%)	0 (0%)	1 (100%)	
**NA**	1	0	1	0	
**Localization**					**.02**
**Hypopharynx**	0 (0%)	0 (0%)	0 (0%)	2 (100%)	
**Larynx**	2 (66.7%)	1 (33.3%)	0 (0%)	0 (0%)	
**Oral cavity and lips**	3 (18.8%)	7 (43.8%)	4 (25%)	2 (12.5%)	
**Oropharynx**	11 (45.8%)	11 (45.8%)	0 (0%)	2 (8.3%)	
**L1**					.36
**absent**	16 (38.1%)	16 (38.1%)	4 (9.5%)	6 (14.3%)	
**present**	0 (0%)	3 (100%)	0 (0%)	0 (0%)	
**Pn1**					.58
**absent**	15 (37.5%)	17 (42.5%)	3 (7.5%)	5 (12.5%)	
**present**	1 (20%)	2 (40%)	1 (20%)	1 (20%)	
**Margin status**					.38
**negative/close**	11 (37.9%)	9 (31%)	3 (10.3%)	6 (20.7%)	
**positive**	2 (25%)	5 (62.5%)	1 (12.5%)	0 (0%)	
**NA**	3	5	0	0	

Supplemental Table S4: Multivariate survival analysis of cellular dissociation grading (CDG) including age, sex, HPV status, localization, AJCC stage, and tumor margin status.

Endpoint	Variable	Hazard ratio	Confidence interval	P
PFI	**CDG (high vs. low)**	**1.55**	**1.05 to 2.28**	**.03**
	**Age (>61 vs. ≤61)**	1.03	0.7 to 1.52	.87
	**Sex (female vs. male)**	1.15	0.74 to 1.8	.53
	**HPV status (pos. vs. neg.)**	1.36	0.69 to 2.66	.37
	**Localization (oropharynx vs. other)**	0.52	0.21 to 1.27	.15
	**AJCC stage (IV vs. I-III)**	**1.9**	**1.22 to 2.93**	**.004**
	**tumor margin status (pos. vs. neg./close)**	**2.48**	**1.57 to 3.92**	**< .001**
OS	**CDG (high vs. low)**	**1.74**	**1.21 to 2.49**	**.003**
	**Age (>61 vs. ≤61)**	**1.46**	**1.02 to 2.08**	**.04**
	**Sex (female vs. male)**	**1.46**	**1 to 2.13**	**.05**
	**HPV status (pos. vs. neg.)**	1.67	0.91 to 3.07	.1
	**Localization (oropharynx vs. other)**	0.82	0.39 to 1.75	.61
	**AJCC stage (IV vs. I-III)**	**1.88**	**1.27 to 2.78**	**.002**
	**tumor margin status (pos. vs. neg./close)**	**1.99**	**1.27 to 3.13**	**.003**

Supplemental Table S5: Multivariate survival analysis of WHO grading including age, sex, HPV status, localization, AJCC stage, and tumor margin status.

Endpoint	Variable	Hazard ratio	Confidence interval	P
PFI	**WHO grade (G2/G3 vs. G1)**	1.18	0.64 to 2.19	.6
	**Age (>61 vs. ≤61)**	0.99	0.67 to 1.46	0.97
	**Sex (female vs. male)**	1.17	0.74 to 1.83	.5
	**HPV status (pos. vs. neg.)**	1.23	0.62 to 2.41	.55
	**Localization (oropharynx vs. other)**	0.48	0.19 to 1.2	.12
	**AJCC stage (IV vs. I-III)**	**1.88**	**1.21 to 2.93**	**.005**
	**tumor margin status (pos. vs. neg./close)**	**2.37**	**1.5 to 3.75**	**< .001**
OS	**WHO grade (G2/G3 vs. G1)**	1.43	0.81 to 2.52	.22
	**Age (>61 vs. ≤61)**	1.36	0.96 to 1.94	.08
	**Sex (female vs. male)**	1.45	0.99 to 2.11	.055
	**HPV status (pos. vs. neg.)**	1.43	0.78 to 2.62	.25
	**Localization (oropharynx vs. other)**	0.75	0.35 to 1.61	.47
	**AJCC stage (IV vs. I-III)**	**1.85**	**1.25 to 2.75**	**.002**
	**tumor margin status (pos. vs. neg./close)**	**1.83**	**1.17 to 2.88**	**.009**

Supplemental Table S6: Multivariate survival analysis of Brandwein-Gensler (BG) score including age, sex, HPV status, localization, AJCC stage, and tumor margin status.

Endpoint	Variable	Hazard ratio	Confidence interval	P
PFI	**BG score (high vs. low/intermediate)**	1.49	1 to 2.23	.051
	**Age (>61 vs. ≤61)**	1	0.67 to 1.49	0.99
	**Sex (female vs. male)**	1.07	0.67 to 1.7	.79
	**HPV status (pos. vs. neg.)**	1.08	0.51 to 2.28	.84
	**Localization (oropharynx vs. other)**	0.58	0.23 to 1.48	.26
	**AJCC stage (IV vs. I-III)**	**1.63**	**1.05 to 2.55**	**.03**
	**tumor margin status (pos. vs. neg./close)**	**2.2**	**1.37 to 3.53**	**.001**
OS	**BG score (high vs. low/intermediate)**	**1.48**	**1.03 to 2.13**	**.03**
	**Age (>61 vs. ≤61)**	1.41	0.98 to 2.03	.06
	**Sex (female vs. male)**	1.31	0.88 to 1.94	.18
	**HPV status (pos. vs. neg.)**	1.6	0.84 to 3.04	.15
	**Localization (oropharynx vs. other)**	0.74	0.35 to 1.6	.45
	**AJCC stage (IV vs. I-III)**	**1.69**	**1.14 to 2.51**	**.009**
	**tumor margin status (pos. vs. neg./close)**	**1.65**	**1.04 to 2.62**	**.03**

Supplemental Table S7: Multivariate survival analysis of cellular dissociation grading (CDG), WHO grading, Brandwein-Gensler (BG) score as well as age, sex, HPV status, localization, AJCC stage, and tumor margin status.

Endpoint	Variable	Hazard ratio	Confidence interval	P
PFI	**CDG (high vs. low)**	1.42	0.94 to 2.14	.09
	**BG score (high vs. low/intermediate)**	1.38	0.91 to 2.09	.12
	**WHO grade (G2/G3 vs. G1)**	1.14	0.61 to 2.13	.68
	**Age (>61 vs. ≤61)**	1.04	0.7 to 1.55	.85
	**Sex (female vs. male)**	1.06	0.66 to 1.69	.81
	**HPV status (pos. vs. neg.)**	1.12	0.53 to 2.37	.76
	**Localization (oropharynx vs. other)**	0.65	0.26 to 1.65	.36
	**AJCC stage (IV vs. I-III)**	**1.62**	**1.03 to 2.54**	**.04**
	**tumor margin status (pos. vs. neg./close)**	**2.29**	**1.42 to 3.69**	**< .001**
OS	**CDG (high vs. low)**	**1.53**	**1.06 to 2.21**	**.02**
	**BG score (high vs. low/intermediate)**	1.37	0.95 to 1.99	.09
	**WHO grade (G2/G3 vs. G1)**	1.35	0.76 to 2.41	.3
	**Age (>61 vs. ≤61)**	**1.53**	**1.06 to 2.21**	**.02**
	**Sex (female vs. male)**	1.3	0.88 to 1.92	.2
	**HPV status (pos. vs. neg.)**	1.67	0.88 to 3.16	.11
	**Localization (oropharynx vs. other)**	0.85	0.4 to 1.83	.68
	**AJCC stage (IV vs. I-III)**	**1.63**	**1.09 to 2.43**	**.02**
	**tumor margin status (pos. vs. neg./close)**	**1.75**	**1.1 to 2.78**	**.02**

A

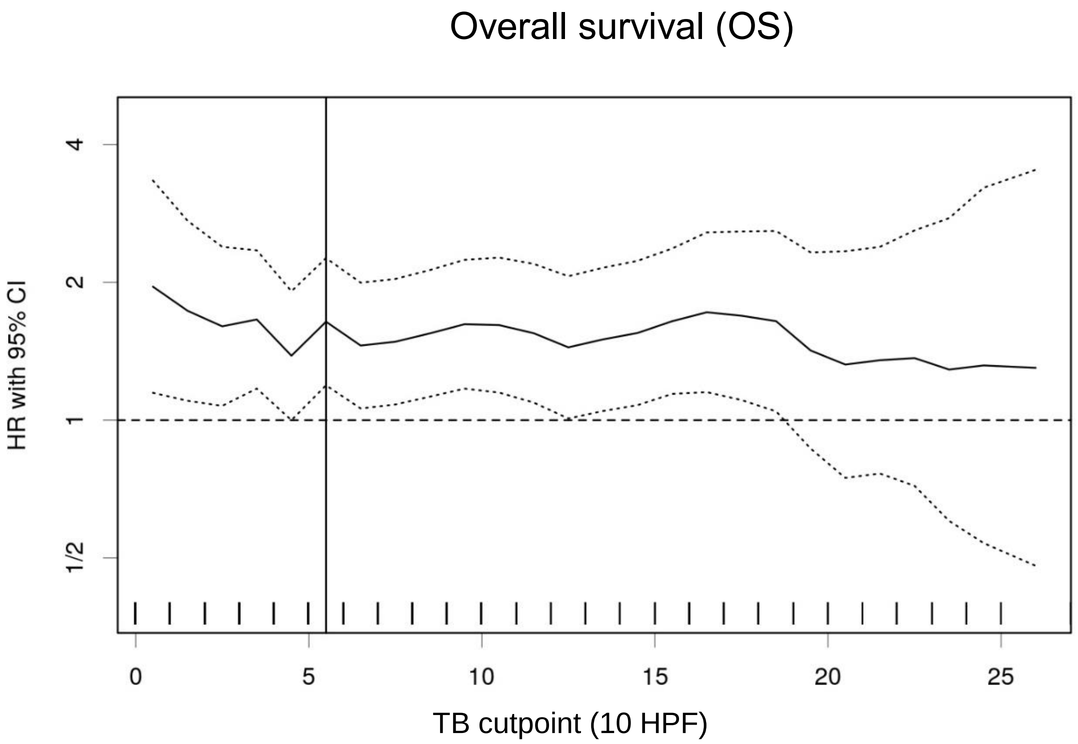

B

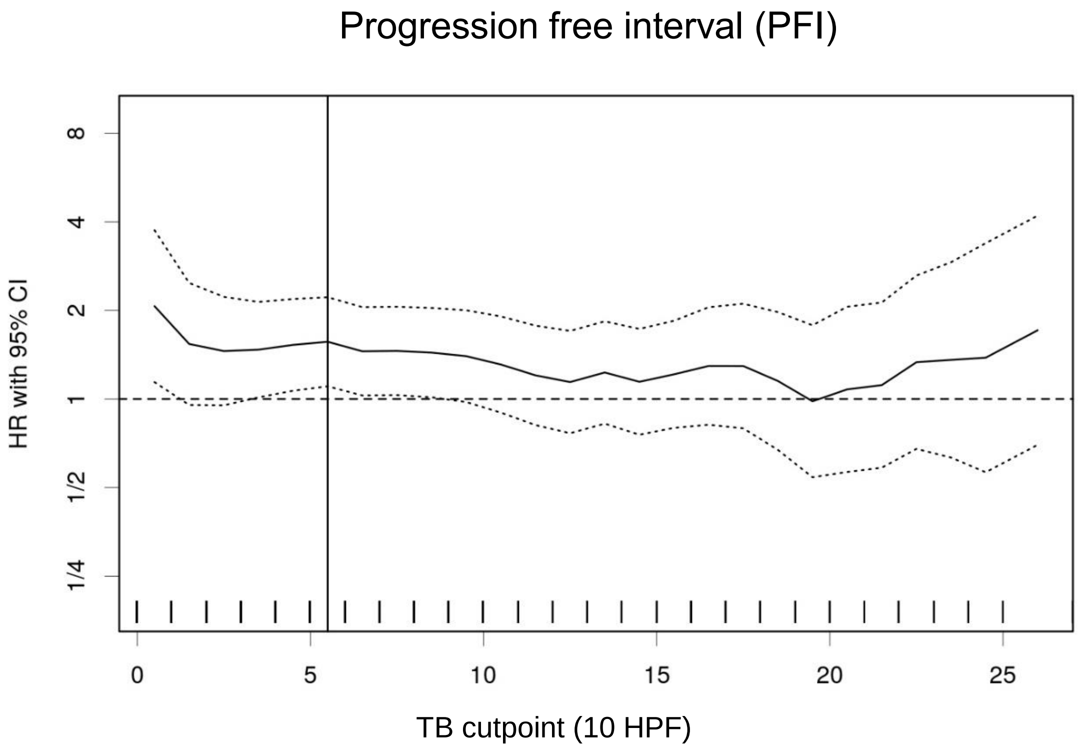

Supplemental Figure S1: Analysis of the prognostic significance of different TB cutpoints (1, …, 27 budding foci). For both, OS **(A)** and PFI **(B)** the most significant difference in survival was achieved by separating tumors with ≥ 6 budding foci from tumors with < 6 budding foci (10 HPF approach).

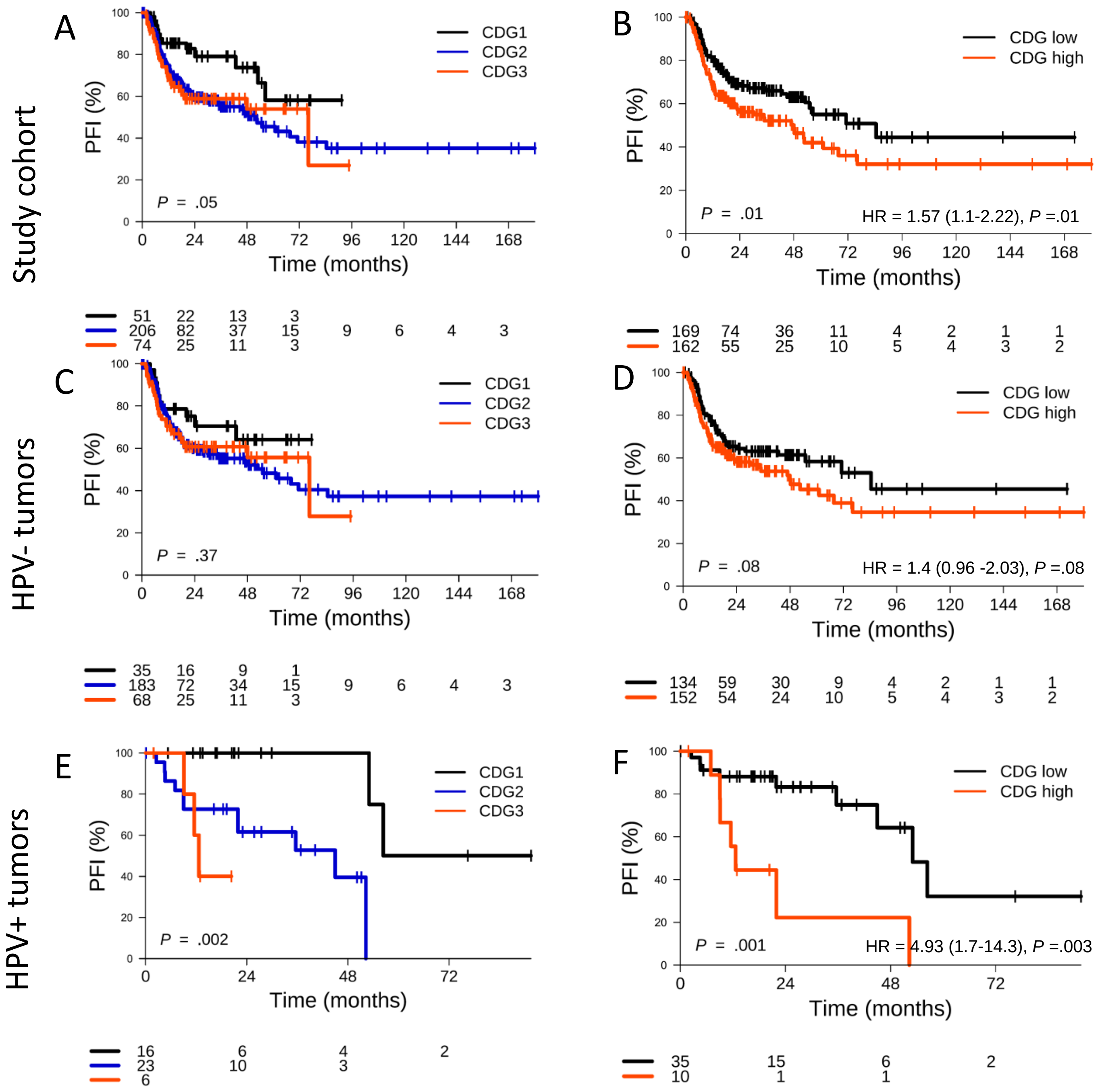

Supplemental Figure S2: Association of progression-free interval (PFI) with cellular dissociation grading (CDG). Performance of the 3-tiered CDG system and of the 2-tiered CDG system in the study cohort **(A/B)**, in the subcohort of HPV-negative tumors **(C/D)** and in the subcohort of HPV-positive tumors **(E/F)**.

A: PFI

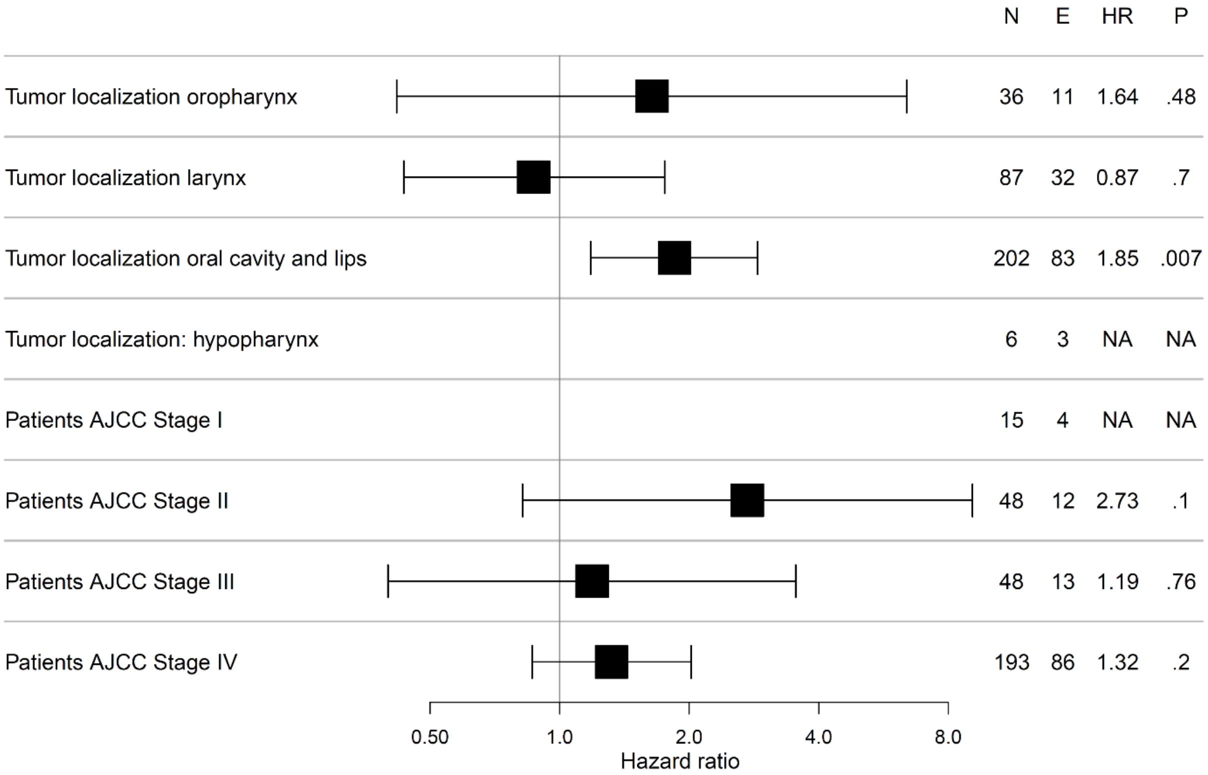

B: OS

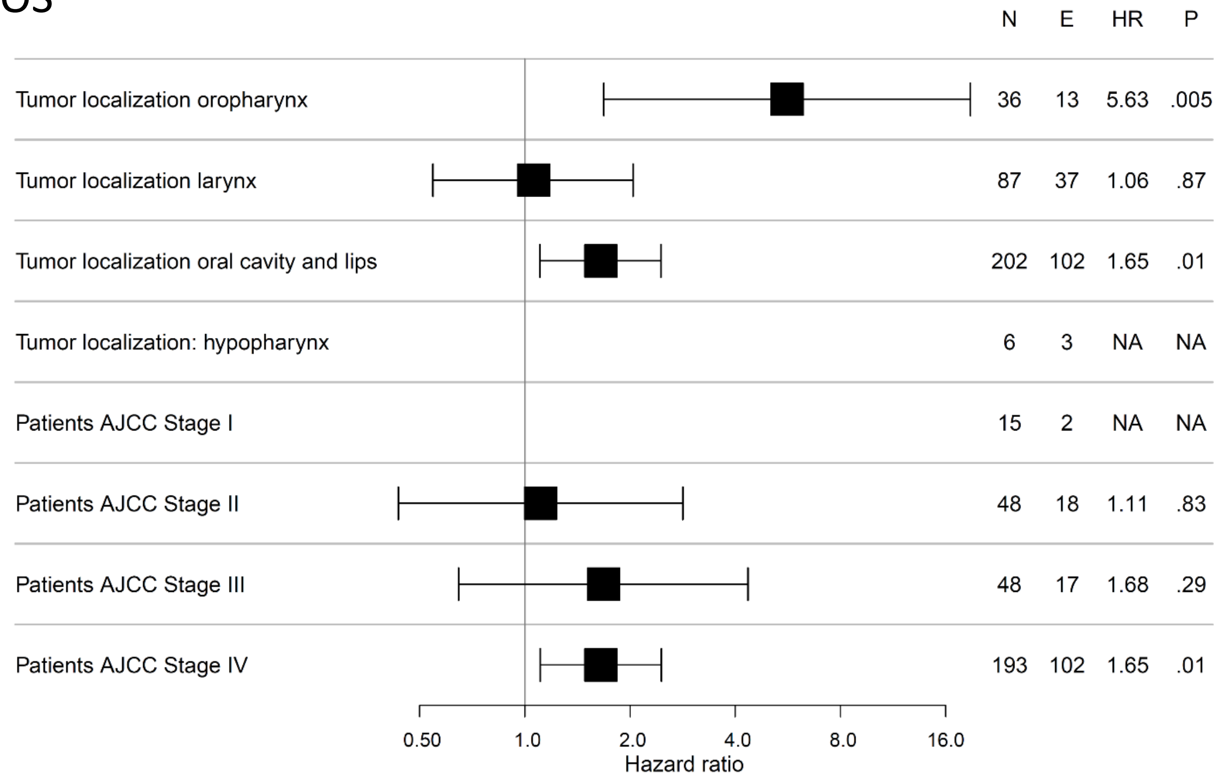

Supplemental Figure S3: Extended subgroup analysis of the prognosticity of cellular dissociation grading (CDG). Comparison of CDG high with CGD low tumors with respect to PFI **(A)** and OS **(B)**. N = number of patients, E = number of events, HR = hazard ratio, CI = 95% confidence interval, NA = not applicable because of too few events.

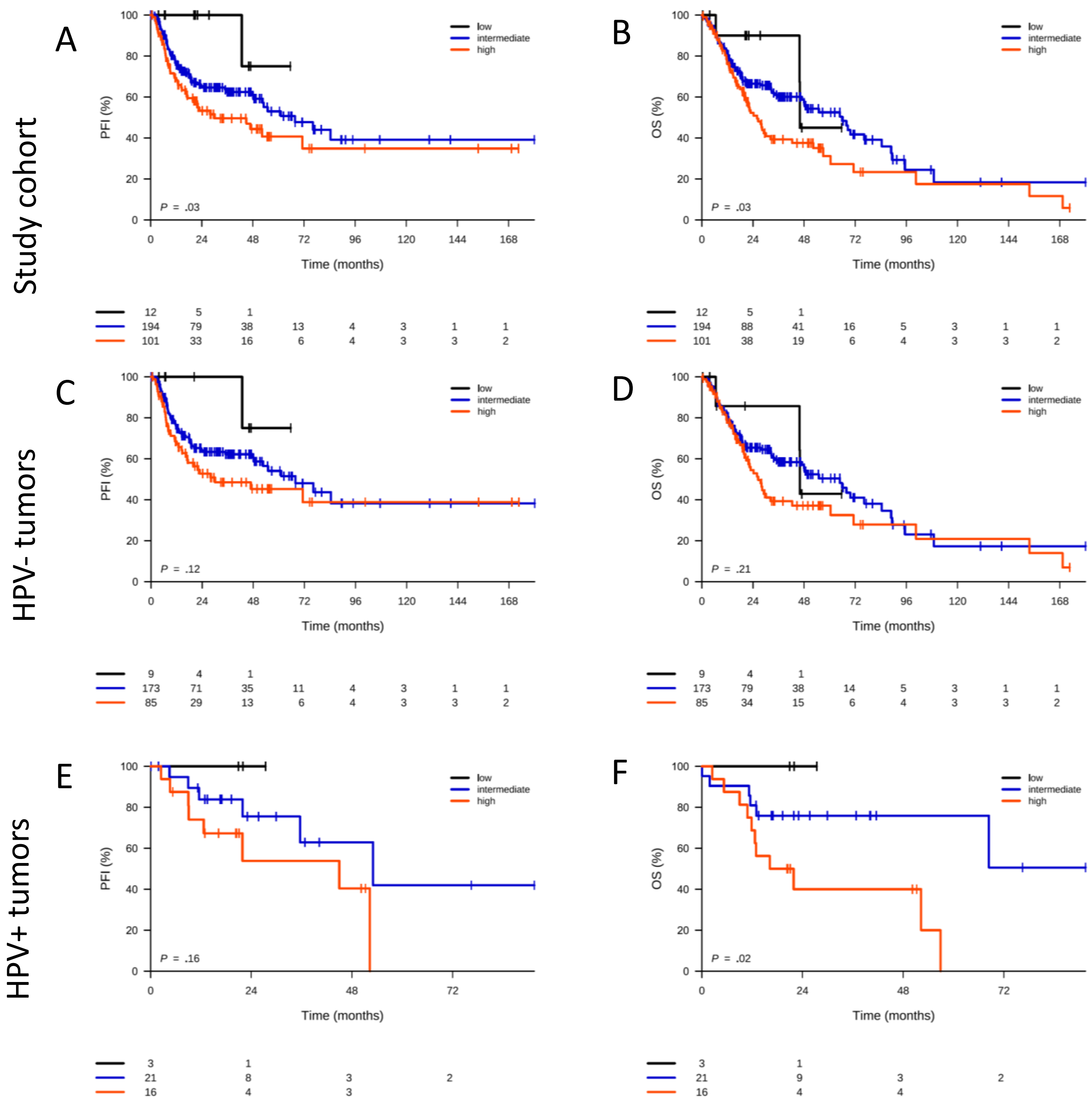

Supplemental Figure S4: Prognostic impact of the Brandwein-Gensler (BG) score. Analysis of PFI and OS in the study cohort **(A/B)**, in the subgroup of HPV-negative tumors **(C/D),** and in the subgroup of HPV-positive tumors **(E/F)**.

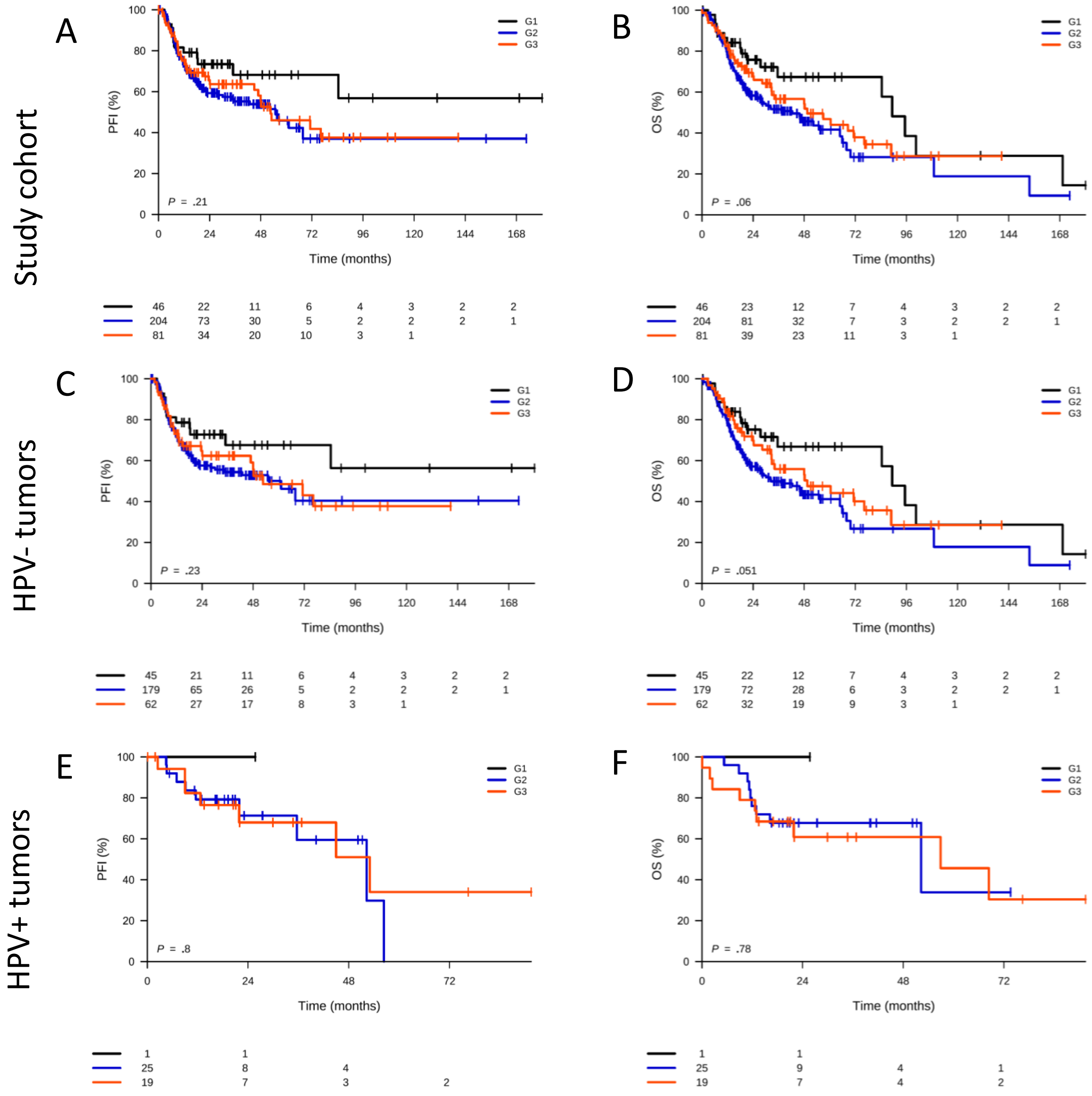

Supplemental Figure S5: Prognostic impact of WHO grading. Analysis of PFI and OS in the study cohort **(A/B)**, in the subgroup of HPV-negative tumors **(C/D),** and in the subgroup of HPV-positive tumors **(E/F)**.

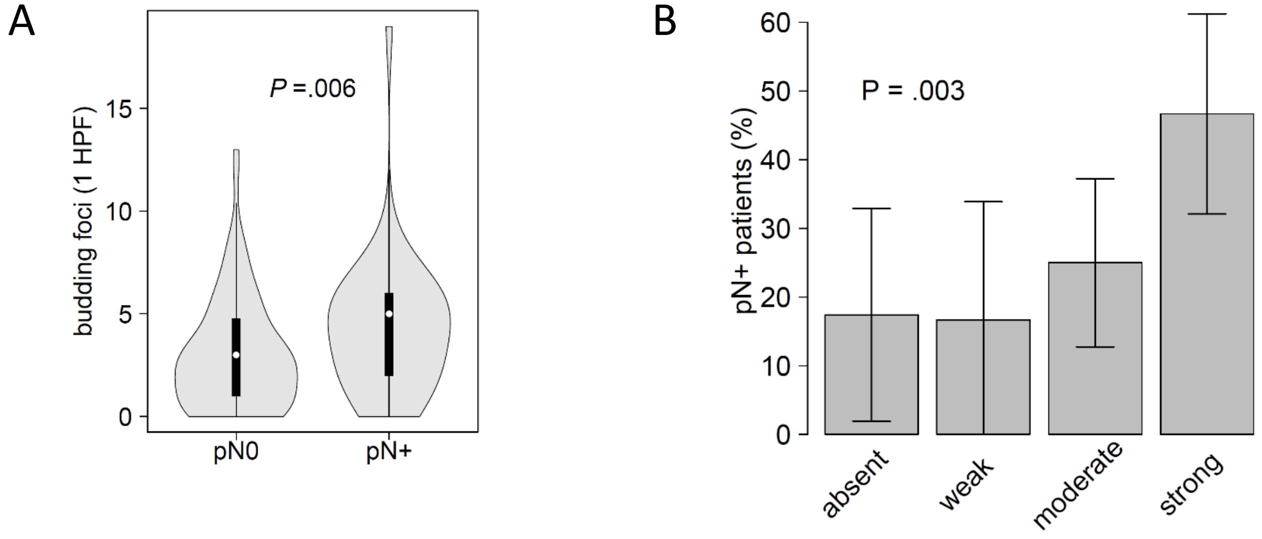

Supplemental Figure S6: Analysis of tumor budding (TB) in 1 high-power field (HPF) of clinically nodal-negative (cN0) patients. A Higher numbers of budding foci (1 HPF) in pathologically nodal-positive (pN+) compared to pN0 patients. **B** Increasing percentages of pN+ patients with increasing TB (1 HPF).
